# Highly Sensitive Love Mode Acoustic Wave Platform with SiO_2_ Wave-Guiding Layer and Gold Nanoparticles for Detection of Carcinoembryonic Antigens

**DOI:** 10.3390/bios12070536

**Published:** 2022-07-18

**Authors:** Chong Li, Jikai Zhang, Haiyu Xie, Jingting Luo, Chen Fu, Ran Tao, Honglang Li, Yongqing Fu

**Affiliations:** 1 Shenzhen Key Laboratory of Advanced Thin Films and Applications, College of Physics and Optoelectronic Engineering, Shenzhen University, Shenzhen 518060, China; 2170218809@email.szu.edu.cn (C.L.); jikai.zhang@northumbria.ac.uk (J.Z.); 1810342119@email.szu.edu.cn (H.X.); luojt@szu.edu.cn (J.L.); chenfu@szu.edu.cn (C.F.); 2 Faculty of Engineering and Environment, Northumbria University, Newcastle upon Tyne NE1 8ST, UK; 3 National Center of Nanoscience and Technology, Beijing 100190, China; lhl@nanoctr.cn; 4 GBA Research Innovation Institute for Nanotechnology, Guangzhou 510530, China

**Keywords:** love wave, biosensors, CEA, AuNPs

## Abstract

A highly sensitive and precise Love wave mode surface acoustic wave (SAW) immunosensor based on an ST-cut 90°X quartz substrate and an SiO_2_ wave-guiding layer was developed to detect cancer-related biomarkers of carcinoembryonic antigens (CEAs). A delay line structure of the SAW device with a resonant frequency of 196 MHz was designed/fabricated, and its surface was functionalized through CEA antibody immobilization. The CEA antibodies were bound with gold nanoparticles and CEA antibodies to form a sandwich structure, which significantly amplified the mass loading effect and enhanced the maximum responses by 30 times. The center frequency of the Love wave immunosensor showed a linear response as a function of the CEA concentration in the range of 0.2–5 ng/mL. It showed a limit of detection of 0.2 ng/mL, and its coefficient of determination was 0.983. The sensor also showed minimal interference from nonspecific adsorptions, thus demonstrating its promise for point-of-care applications for cancer biomarkers.

## 1. Introduction

Cancer is regarded one of the dominant health issues worldwide and also one of the leading causes of high death rates [1]. Its cure rate becomes very low once passing its early stages [2,3,4]. Early cancer detection has been crucial for its treatment. One of the commonly observed clinical tumor markers for screening, e.g., the carcinoembryonic antigen (CEA), has been applied for the diagnosis of tumors including breast [5], colon [6], lung [7], pancreatic [8], cervical and ovarian tumors [9]. It is essential to develop rapid and precision detection of the CEA for the early diagnosis and treatment of cancers. Currently, there are various detection methods for the CEA that have been developed, for example, the radiation immunological assay [10], fluorescence immunoassay [11], enzyme-linked immunosorbent assay (ELISA) [12], electrochemical immunoassay [13,14], surface-enhanced Raman scattering (SERS) immunoassay [15], chemiluminescence immunoassay [16] and piezoelectric immunosensors [17]. These methods have their advantages including high sensitivity and specificity due to their specificity and affinity between the antigen and antibody in the sensing process. 

Piezoelectric-based immunosensors are widely reported in medical applications, such as the detection of bacteria, viruses and toxins [18], and clinical diagnosis [19]. Sensors based on surface acoustic waves (SAWs) are often regarded as mass-sensitive detectors for label-free immunoassays because of there numerous advantages [20], including wireless capability [21], real-time monitoring [22], high selectivity [23], good biocompatibility [24] and simplicity of use [25]. In particular, shear horizontal (SH) SAW sensors and Love wave sensors have shown their advantages due to their outstanding stability and sensitivity in liquid environments [26,27,28,29]. Love wave-based sensors commonly consist of a wave-guiding layer (which has a low shear wave velocity or a low density) deposited onto an SH-SAW substrate. In order to enhance the sensitivity of the Love mode sensor, this wave-guiding layer should be optimized, and its elastic properties should be improved to avoid severe dissipation of the acoustic waves. It should confine the acoustic energy within the sensing layer, which can enhance its sensitivity to the corresponding mass loadings on the sensor’s surface and protect the interdigital transducers (e.g., IDTs) from the complex liquid solution. Several types of guiding layers are commonly reported, e.g., SiO_2_ [30,31], polymethylmethacrylate (PMMA) [32,33,34] or photoresist [35], and a multilayer of SiO_2_ and PMMA [36]. With a low shear velocity of about 2850 m/s [33,37], SiO_2_ thin film has good mechanical and thermal properties, and is frequently used as the wave-guiding layer [38]. This layer was further combined with gold nanoparticles (AuNPs) and thus amplified the mass loading effect of the sensor [39], which was extensively used for gas sensing [40] or biosensing (e.g., bacteria [41] or protein detection [42] in a liquid environment).

Recently, there have been many reports about the various methods for detecting disease-related biomarkers, including cancer detection. The optical biosensing methods have been widely applied to detect disease-related biomarkers, but the significant interference from background noises, their high cost and complicated operation during detection are the key challenges for their successful commercialization [43]. Quartz crystal microbalance (QCM), one of the key bulk acoustic wave-based sensing platforms, can achieve a large value of limit of detection (LOD), but it has a much lower operating frequency, which is commonly linked with a lower sensitivity according to the Sauerbrey equation [44]. Another bulk wave-based acoustic sensing technique, e.g., film bulk acoustic resonators, has much higher frequencies (in GHz level); however, they are difficult and expensive to manufacture [45]. SAW sensors, especially Love wave mode-based SAW sensors have been applied in biosensing, such as for detecting cancer markers, protein, bacteria, viruses and spores in various liquid environments [46], with reasonable costs and fast, portable, wireless and real-time detection possibilities. It was reported that the thickness/dimension of the wave-guiding layer greatly influences the sensitivity of these Love wave sensors, and finite element analysis (FEA) has often been used to tune the optimized layer thickness and achieve the desired sensing performance [46]. Recently, analytical studies have also been performed to optimize the properties of this wave-guiding layer for Love wave devices [47]. Therefore, a Love wave device based on an SiO_2_ wave-guiding layer on a quartz SAW device will be promising for biosensors applied in complex liquid environments [48].

In this paper, a Love wave mode-based immunosensor was developed for CEA detection using an SiO_2_ wave-guiding layer deposited on an ST-cut 90°X quartz. To improve the sensitivity of this sensor, we applied gold nanoparticle (AuNPs) antibody conjugates on the surface of the SAW device to enhance the sensing performance for the conventional sandwich immunoassay. We further optimized the thickness of the wave-guiding layer (SiO_2_) using both FEA analysis and experimental studies. Finally we evaluated the sensing performance of the devices, including their sensitivity, LOD and specificity, at different CEA concentrations.

## 2. Methods

### 2.1. Finite Element Analysis

To study thickness effects of SiO_2_, FEA was performed using the commercial software COMSOL with modules of solid mechanics and electrostatics. The 3D model consisted of a 100-μm-thick quartz substrate and SiO_2_ wave-guiding layers with different thicknesses from 200 nm to 1600 nm. Cross-section of the 2D model was rectangle, i.e., 1 μm × 25 μm. Pairs of gold electrodes were applied as the electrical ground and floating potential (10 V) nodes with a width of 6.25 μm, corresponding to the designed wavelength of 25 μm. Free tetrahedral meshes were built for both quartz substrate and SiO_2_ guiding layer. The mesh was mainly refined for the model within a wavelength depth on the surface, while the mesh near the bottom was coarsened. Vibration patterns, wave modes, surface displacements and resonant frequency were simulated using the eigenfrequency analysis method available in the COMSOL.

Continuous periodic boundary conditions were used to optimize the model as below

(1) U(v)=U(v+λ)

where $U\left(v\right)=\left[\begin{array}{c} u\left(v\right)\\ \phi \left(v\right) \end{array}\right]$, λ is the wavelength of IDT electrodes, u(v) is the motion equation of the boundary and ϕ(v) is the potential of the boundary. A normalized layer thickness was defined as the ratio between thickness of SiO_2_ guiding layer (h) and the designed wavelength (λ). Mass sensitivity of the Love mode SAW sensor was calculated using the following equation [49]

(2) $$S=\underset{∆m\to 0}{\lim}\frac{1}{∆m}\frac{∆f}{f_{0}}=\underset{∆m\to 0}{\lim}\frac{1}{∆m}\frac{∆v}{v_{0}}$$

where ∆m is the change in mass on the device surface, $v_{0}$ is the acoustic velocity and $f_{0}$ is device’s center frequency, ∆v and ∆f represent the shifts of the acoustic velocity and center frequency after the target material was immobilized, respectively.

### 2.2. Fabrication Process of SAW Device

A four-inch ST-cut and single-side polished quartz wafer (500-μm-thick) was rinsed by acetone, ethanol and deionized water sequentially, and then dried using N_2_ gas. Metallic IDTs were fabricated via a conventional photolithographic lift-off process, and Ti/Au (10-nm-/100-nm-thick) layers were patterned on the quartz substrate using the electron beam evaporation. The IDTs consisted of 50 pairs of fingers with 25 μm wavelength and 6.25 μm width. The delay line had 3 mm width and 7 mm length. 

The SiO_2_ guiding layers with different thicknesses varying from 200 nm to 1580 nm were patterned on the SAW device using a magnetron sputter with the contact pads protected by the Kapton tape. A DC power of 150 W was used with a sputtering pressure set to be 0.5 Pa, and the gas volume ratio of Ar:O_2_ was 1:1. The crystalline structure of the deposited SiO_2_ was characterized using X-ray diffraction (XRD, Rigaku, Japan). Surface and cross-sectional morphologies of the SiO_2_ layers were acquired using a scanning electron microscope (SEM, Hitachi S-800, Japan). The obtained results are shown in the supplementary information Figures S2 and S3.

### 2.3. Preparation of AuNPs–Antibody Conjugates

AuNPs were synthesized by mixing and reaction of hydrochloroauric acid and sodium citrate [50]. The size of AuNPs was estimated from the SEM observation. The CEA antibody solution was added to phosphate-buffered saline (PBS) buffer to prepare a 100 µg/mL solution. CEA antibody solution with a volume of 400 μL was mixed with 2 mL of AuNP’s solution under a gentle stirring. This process was followed by incubation for 60 min, during which the antibodies were adsorbed onto the AuNPs through electrostatic process. Then the obtained solutions were centrifuged at a speed of 10,000 rpm for 20 min. After removing clear supernatant liquid by using centrifugation, the collected precipitate was then dispersed into 1000 mL PBS with 0.1% Bovine Serum Albumin (BSA). Finally, the AuNP–antibody conjugate solution was stored at 4 °C for further experiments.

### 2.4. Surface Functionalization of the Love Wave Sensor

Surface of SAW sensor was cleaned using solvents and treated by O_2_ plasma, followed by soaking in 20 mM APTES (with the solvent of pure ethyl alcohol) for 30 min. The device was heated to 110 °C in an oven and kept for 30 min. It was rinsed by ethyl alcohol and then dried using N_2_ gas. The sensor was then kept at room temperature and soaked in 20 mM terephthalaldehyde for 30 min, which was followed by rinsing with deionized water and dried using N_2_ gas. Contact angles and hysteresis of the sessile droplets were acquired using a droplet shape analyzer after each process. The CEA antibody (from 1 μL of 1 mg/mL solution) was captured onto the surface of the Love wave sensor and incubated for 12 h. Uncaptured CEA antibodies were removed by rinsing the device with the PBS buffer. After drying with N_2_ gas, the Love wave sensor was immersed in 200 mL BSA/0.1% PBS for 90 min and then washed with PBS buffer and dried using N_2_ gas. To verify if the captured CEA antibody was successfully bound onto the device’s surface after functionalization, fluorescent capture CEA antibodies were used in the treatments described above, and the obtained images observed under a fluorescence microscope (ECLIPSE Ni-U, Nikon, Tokyo, Japan) are shown in the supplementary information Figure S4. The prepared Love wave biosensor was then stored at 4 °C.

### 2.5. Biosensing System

**Figure 1:**
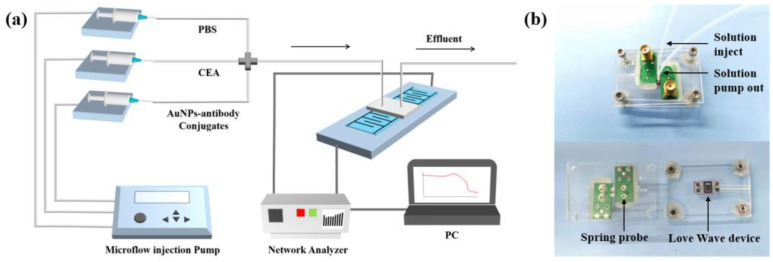
(**a**) Schematic illustration of the CEA measurement system; (**b**) photos of the detection platform based on the SAWs and the PDMS microchannel.

The biosensing system consists of three modules, i.e., the flow injection pumps, detection platform and signal processing units. Figure 1 shows flow injection module (TS-1B/4*W0109-1B, 40W, Longer company, Baoding, China), which was used to inject the buffer and sample solution into a microchannel bonded onto the surface of the sensor using a flow rate of 16.7 μL/min. In order to reduce the interference caused by changing the different solutions during the experiment, a chamber with three channels connected with three microflow injection pumps was fabricated with polydimethylsiloxane (PDMS). The detection was performed using the SAW sensor and a PDMS chamber as shown in Figure 1b. A rubber ring was pressed on the sensitive area of the Love Wave device, and two spring probes were connected to the bottom acrylic plate, The spring probe was in tight contact with the two electrodes of the device. The acrylic plate was fixed by four nuts in order to support microfluidics and avoid the liquid spillover. The frequency shifts at the resonant frequency of 196 MHz were recorded using a network analyzer (E5701C, Agilent Technologies, Santa Clara, CA, USA) to reveal the mass loading on the sensor. Two Positive Intrinsic Negative (PIN) diode switches were placed between the sensor and the network analyzer for continuous measuring. Data were conducted from the network analyzer using LabVIEW software specifically programmed for this experiment.

### 2.6. Detection Strategy of CEA

The sensing areas of the biosensor were modified by the PDMS structure as the microfluidic channel, so that the sensing was able to operate in a liquid environment. Firstly, 1 mL PBS with a speed of 1 mL/h was injected into the chamber and the liquid flowed through the sensing area until a stable signal was obtained. For the direct immunoassay without using AuNPs, the captured anti-CEA antibodies were pre-patterned onto the device’s surface, and then specifically bound with the CEAs. The capture of CEAs was followed by the addition of AuNP–antibody conjugates, which contained the anti-CEA antibodies specifically bound with CEAs.

## 3. Results and Discussion

### 3.1. Optimized Parameters of Wave-Guiding Layer

**Figure 2:**
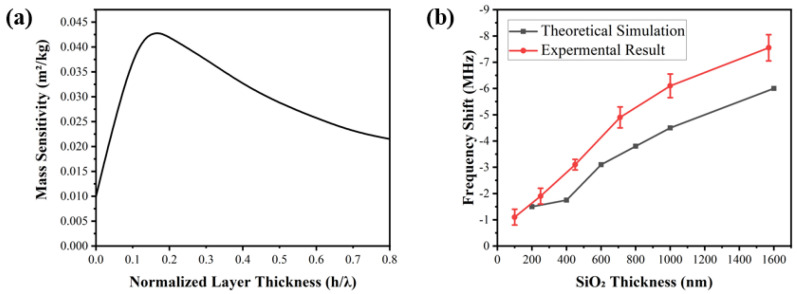
(**a**) Mass sensitivity of the Love mode SAW device as a function of the normalized layer thickness in the sensing system; (**b**) comparisons between experimental results and theoretical sim −ulation results with various SiO_2_ thicknesses.

Figure 2a shows the relationship between the normalized thickness of the wave-guiding layer and the mass sensitivity of the SAW device, which were obtained from the FEA results. The device’s sensitivity increases with the increase in the normalized thickness of the wave-guiding layer until reaching the maximum value at a normalized value of 0.14 as shown in Figure 2a. After this, the mass sensitivity was found to decrease with the normalized thickness of the wave-guiding layer. Based on the results, we can conclude that the optimal thickness of this wave-guiding layer is estimated to be ~3.5 μm according to the computational simulations. As the thickness of the wave-guiding layer increases, the SAW energy is gradually confined onto the device’s surface because the shear velocity value of the SiO_2_ layer is much lower than that of ST-quartz. Therefore, this results in an enhanced mass sensitivity, increased center frequency and decreased insertion loss, all of which improve the sensing performance of the sensor.

Figure 2b shows the shifts in the frequency values of the SAW device with the increase in SiO_2_ thickness, compared to those without a wave-guiding layer. The slight differences in the selected thickness values between the experiment data and simulation data are mainly from the instrumental errors of the film thickness control/monitoring during the sputtering deposition. Both the experimental and simulation results show the same tendency where the variation in the center frequencies decreases slowly when the thickness of the SiO_2_ layer is decreased to the optimal thickness obtained from the simulation. Experimental results show that the shifts in center frequencies for the sensors are 6 MHz and 7.56 MHz, with the SiO_2_ thicknesses of 1000 nm and 1580 nm, respectively. The corresponding changes in the theoretical simulation results are around 4.65 MHz and 6.06 MHz, respectively. Normally, during FEA simulations, we assumed perfect material structures, default material properties, a good match between the SiO_2_ and quartz layers, and zero impedance mismatch with the external circuits. However, the sputtered SiO_2_ films possess defects and interfacial stresses generated due to the lattice mismatch. Therefore, the experimentally obtained acoustic velocity of SiO_2_ films was smaller compared to that of the ideal material in the simulation, hence resulting in a larger frequency change.

**Figure 3:**
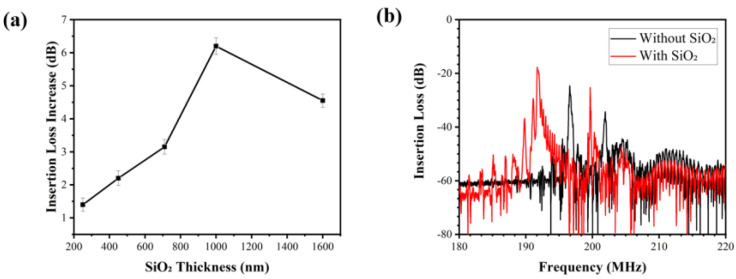
(**a**) The variation in insertion losses of the SAW device with various thicknesses of SiO_2_ (three repetitions were performed); (**b**) S21 transmission spectra for the SAW device with and with −out 1000-nm-thick SiO_2_.

From Figure 2, the 1580-nm-thick SiO_2_ wave-guiding layer shows the best effect on the changes in the center frequency of the sensor. However, it is not the optimal thickness when considering the effect of the various SiO_2_ thicknesses on insertion losses of the sensor. Figure 3a shows that with the increase in SiO_2_ thickness, the insertion loss of the sensor firstly increases but then decreases. The maximum value was found at the wave-guiding layer thickness of 1000 nm. The insertion loss of the 1000 nm SiO_2_ wave-guiding layer thickness is −17 dB obtained by measuring the S21 signal of the sensor (as shown in Figure 3b). An SiO_2_ layer 1000-nm-thick was eventually selected as the optimized layer for reducing wave damping in a liquid environment, although it might cause a decrease in the center frequency and its insertion loss. 

### 3.2. Characterization of AuNPs and Surface Functionalization

**Figure 4:**
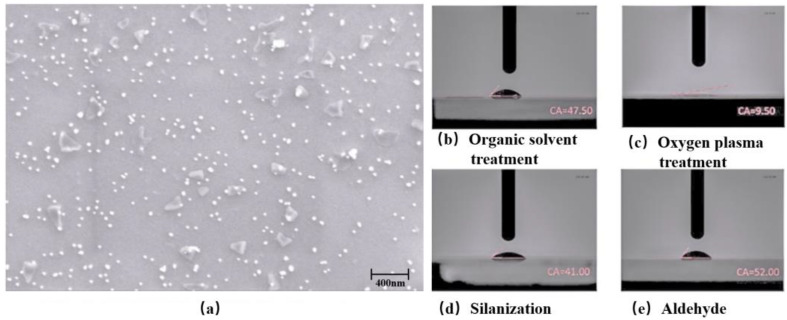
(**a**) SEM image of AuNPs with the magnification of 25,000×. Contact angles of the sensor surface after (**b**) organic solvent treatment, (**c**) oxygen plasma treatment, (**d**) salinization and (**e**) aldehyde treatment.

Figure 4a shows that the AuNPs are homogeneously dispersed with spherical patterns (~20 nm in diameter). Figure 4b shows the contact angles on the Love wave sensor’s surface after each fabrication process. The hydrophilicity of the sensor is decreased after the aldehyde–salinization treatment, which proves that the surface functionalization has successfully created the aldehyde groups on the surface of the sensor. These aldehyde groups were further used to bind the amidogen groups of the captured CEA antibody.

### 3.3. Sensing Performance for CEA Detection

**Figure 5:**
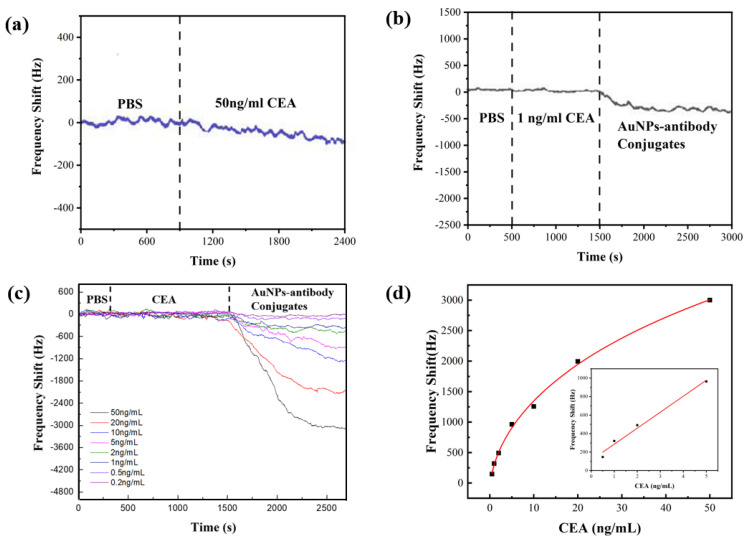
(**a**) Real-time responses of Love mode sensor to 50 ng/mL CEA in the immunoassay with −out AuNPs. (**b**) Real-time responses of the sensor to 1 ng/mL CEA in the AuNP simplified sand −wich immunoassay. (**c**) Real-time responses of sensor to CEA with the concentration varying from 0.2 ng/mL to 50 ng/mL. (**d**) Measured frequency shifts as a function of CEA concentration.

Real-time frequency shifts of the SAW sensors were recorded during the sensing process. Figure 5a shows the sensor’s responses of the immunoassay without using AuNPs, where the PBS solution flows over the sensor chip for 15 min to evaluate the background noise level, followed by flowing with the CEA solution with the concentration of 50 ng/mL. The center frequency of the sensor decreased slightly due to the bonding of CEA molecules, and then reached a stable value after 30 min. The frequency shift was about 100 Hz, tested with 50 ng/mL CEA, which was similar in magnitude to the background noise of the sensor. 

Our results clearly showed the real-time responses to the AuNP–antibody conjugates were apparently amplified compared to those of the immunoassay without using the AuNPs. Figure 5b shows the results when 1 ng/mL CEA was introduced after the injection of the PBS solution. The CEAs were bound with CEA antibodies within 20 min. However, there were no apparent frequency shifts as the concentration of the CEA was lower than the LOD of the sensor, whereas for the case with the AuNP–antibody added, after 30 min, a frequency shift of around 311 Hz was obtained. The sensing results confirm that the AuNP–antibody conjugates amplify the mass loading effect.

Figure 5c shows the real-time frequency shifts using the CEA samples with their concentrations varied between 0.2 ng/mL and 50 ng/mL. The frequency shifts were nearly negligible during the injection of CEA, but they became obvious when the AuNP–antibody conjugates were added. The absolute value of the frequency shift increased with the increased CEA concentrations and reached a saturation value after injection for 20 min. The calibration curve for the CEA detection is shown in Figure 5d. The frequency shift increased with the CEA concentration, and had a logarithmical relationship with the concentration of CEAs in the range from 0.2 ng/mL to 5 ng/mL. The value of the regression coefficient (R^2^) was found to be 0.98288. However, the relative frequency shift did not follow the logarithmic trend when the CEA concentration ranged from 0.2 ng/mL to 50 ng/mL. This can be explained by the fact that there is a limited area on the surface of the SAW device, thus the amount of the captured CEA antibodies fixed on the sensor surface is limited. The frequency shifts become saturated at a certain point where the CEA concentration is relatively high.

We further estimated the theoretical LOD of the sensor for the CEA solution, using the least-square method to fit the linear regime [51]. About 1800 data points were used after taking consideration of the initial baseline. A standard deviation (S) of 757.31 Hz was obtained from the baseline. The sensor noise (RMS_noise_) was estimated as 17.85 Hz using the root-mean-square deviation (RMSD) following the Equation (3). The theoretical LOD was calculated to be 0.291 ng/mL according to Equation (4).

(3) $${\mathrm{RMS}}_{\mathrm{noise}}=\sqrt{\frac{S^{2}}{N}}.$$

(4) $$\mathrm{LOD}=3\frac{{\mathrm{RMS}}_{\mathrm{noise}}}{\mathrm{Slope}}.$$

where N is the number of data points. This AuNP–antibody conjugate’s amplified assay significantly lowered the value of the LOD when compared to the immunoassay without AuNPs.

### 3.4. Interferences of Nonspecific Adsorptions

Selectivity or specificity is a key parameter for the detection of target biomolecules. In reality, the solution to be mezasured has a complex background including the targeted CEA and a number of biomolecules. The biomolecules could be captured nonspecifically and then interfere with the sensor’s responses. These nonspecific adsorption phenomena were investigated in this study to verify the specificity of the biosensor to CEA. L-tryptophan and Alpha-fetoprotein (AFP), which have a similar functional group compared to CEA, were used as interfering molecules for the background. The sensor was used to perform a nonspecific adsorption test on each of the 50 ng/mL standard solutions separately, and then immobilized with the CEA antibody. Each experiment was repeated three times. 

**Figure 6:**
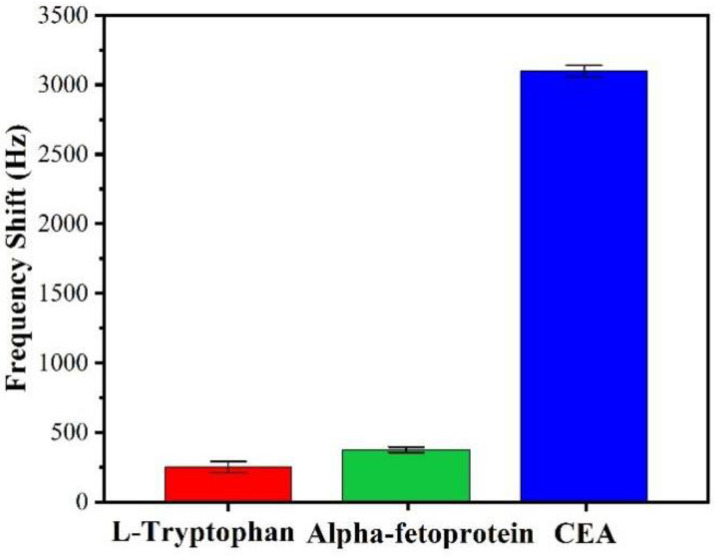
Comparison of the responses of the SAW sensor to 50 ng/mL solutions of L-tryptophan, Alpha-fetoprotein and CEA.

Figure 6 shows the frequency shifts of the Love mode sensor to L-tryptophan, AFP and CEA are 245 ± 17 Hz, 366 ± 21 Hz and 3098 ± 45 Hz, respectively. Clearly the response to the targeted CEA is 10 times stronger than those to L-tryptophan and AFP, indicating that this newly developed SAW immunosensor is apparently unaffected by the interference from the background solutions, thus showing its good selectivity or specificity.

## 4. Summary

A microfluidic SAW immunosensor was studied in this paper using 1000-nm-thick SiO_2_ as the wave-guiding layer and AuNPs as the enhancing agent. The method for detecting CEA is label free and conducted in real time, with high sensitivity and specificity. The best limit of detection obtained was 0.2 ng/mL. The minimal interference of nonspecific adsorptions was verified. Further work will be focused on the catalytic efficiency of the different diameters of AuNPs and the influence of the incubating period of the CEA solution on the biosensor.

## Data Availability

Not applicable.

## Supplementary Materials

The following supporting information can be downloaded at: https://www.mdpi.com/article/10.3390/bios12070536/s1, Figure S1. (a) 3D model of the Love wave sensor; (b) free tetrahedral and mapped meshes applied in the simulation; Figure S2. XRD pattern of the sputtered SiO_2_ thin film; Figure S3. Image of sputtered SiO_2_ of the Love wave device under SEM, (a) surface morphology; (b) cross-sectional views; Figure S4. Comparison of bound fluorescent capture CEA antibody and unbound region. Table S1. Materials and their main properties used in FEA simulations.
