# Supplementary material for: Highly Sensitive Love Mode Acoustic Wave Platform with SiO2 Wave-Guiding Layer and Gold Nanoparticles for Detection of Carcinoembryonic Antigens
Source: Biosensors (Basel). 2022 Jul 18;12(7):536. doi: 10.3390/bios12070536 (PMC9313398; doi:10.3390/bios12070536)
Supplement: Supplementary file 1 [file biosensors-12-00536-s001.zip › biosensors-1765468-supplementary.pdf]

Supplementary information

for

Highly sensitive Love mode acoustic wave platform with SiO2 wave-guiding layer and gold nanoparticles for detection of carcinoembryonic antigens

Chong Li [1,†], Jikai Zhang [1,2,†], Haiyu Xie [1,†], Jingting Luo [1], Chen Fu [1], Ran Tao [1,*], Honglang Li [3,4] and Yongqing Fu [2,*]

1 Shenzhen Key Laboratory of Advanced Thin Films and Applications, College of Physics and Optoelectronic Engineering, Shenzhen University, Shenzhen 518060, China; 2170218809@email.szu.edu.cn (C.L.); jikai.zhang@northumbria.ac.uk (J.Z.); 1810342119@email.szu.edu.cn (H.X.); luojt@szu.edu.cn (J.L.); chenfu@szu.edu.cn (C.F.)

2 Faculty of Engineering and Environment, Northumbria University, Newcastle upon Tyne NE1 8ST, UK

3 National Center of Nanoscience and Technology, Beijing 100190, China; lhl@nanoctr.cn(H.L.)

4 GBA Research Innovation Institute for Nanotechnology, Guangzhou 510530, China.

* Correspondence: ran.tao@szu.edu.cn (R.T.); richard.fu@northumbria.ac.uk (Y.F.)

† These authors contributed equally to this work.

1. Detailed information about FEA simulations

Figure. S1 shows the 3D model of the Love wave sensor and the meshes applied. The Love wave propagates along x axis and the particles oscillate in y direction. Periodic boundary conditions are applied to Γ_B and Γ_F, as well as the other two lateral walls perpendicular to x axis. Fixed constraint is applied to Γ_D.

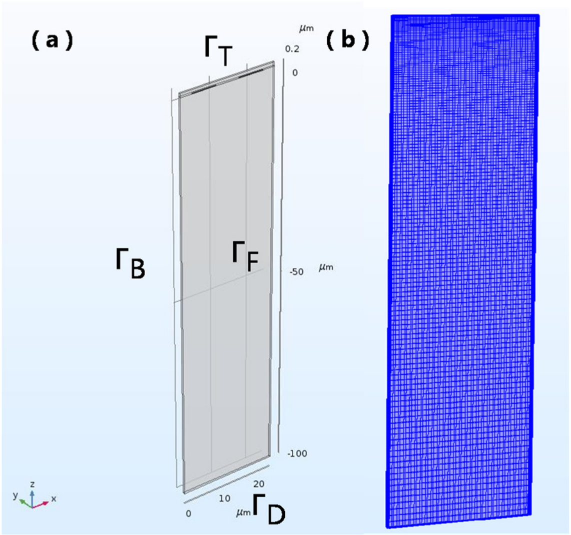

Figure S1 (a) 3D model of the Love wave sensor; (b) Free tetrahedral and mapped meshes applied in the simulation.

In the FEA simulations, we used rotated system with Euler angles of (90°, 132.75°, 0°) to perform the ST-cut 90°X orientation. Therefore, the material properties of standard Quartz LH (1978 IEEE) were used in the Materials section. Table SI1 lists the

important properties of materials used in this work.

Table S1 Materials and their main properties used in FEA simulations

Materials	Material properties
Quartz LH (1978 IEEE)	Elasticity matrix: $[c] = \begin{bmatrix} 86.74 & 6.99 & 11.91 & 17.91 & 0 & 0 \\ 6.99 & 86.74 & 11.91 & -17.91 & 0 & 0 \\ 11.91 & 11.91 & 107.2 & 0 & 0 & 0 \\ 17.91 & -17.91 & 0 & 57.94 & 0 & 0 \\ 0 & 0 & 0 & 0 & 57.94 & 17.92 \\ 0 & 0 & 0 & 0 & 17.92 & 39.91 \end{bmatrix} GPa$
	Coupling matrix: $[e] = \begin{bmatrix} -0.17 & 0.17 & 0 & -0.04 & 0 & 0 \\ 0 & 0 & 0 & 0 & 0.04 & 0.17 \\ 0 & 0 & 0 & 0 & 0 & 0 \end{bmatrix} C/m^2$
	Relative permittivity: $[\varepsilon] = \begin{bmatrix} 4.43 & 0 & 0 \\ 0 & 4.43 & 0 \\ 0 & 0 & 4.63 \end{bmatrix}$
	Density: $\rho = 2651\,kg/m^3$
SiO_2	Young's modulus: $E = 70GPa$
	Poisson's ratio: $\nu = 0.17$
	Relative permittivity: $\varepsilon = 4.2$
	Density: $\rho = 2200\,kg/m^3$
Au	Electrical conductivity: $\sigma = 45.6 \times 10^6\,S/m$

2. Characterization of SiO_2 guiding layers

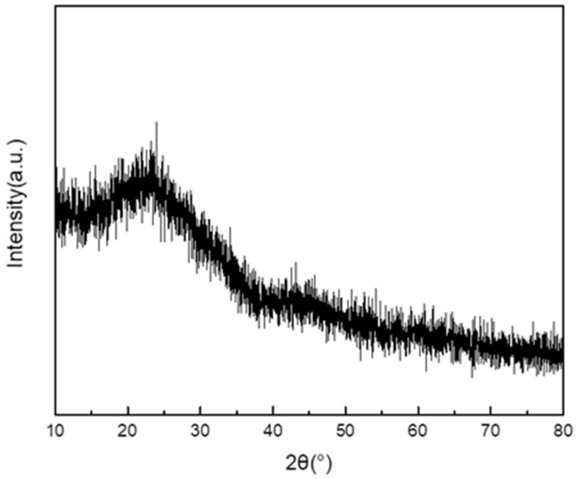

Figure. S2. XRD pattern of the sputtered SiO_2 thin film.

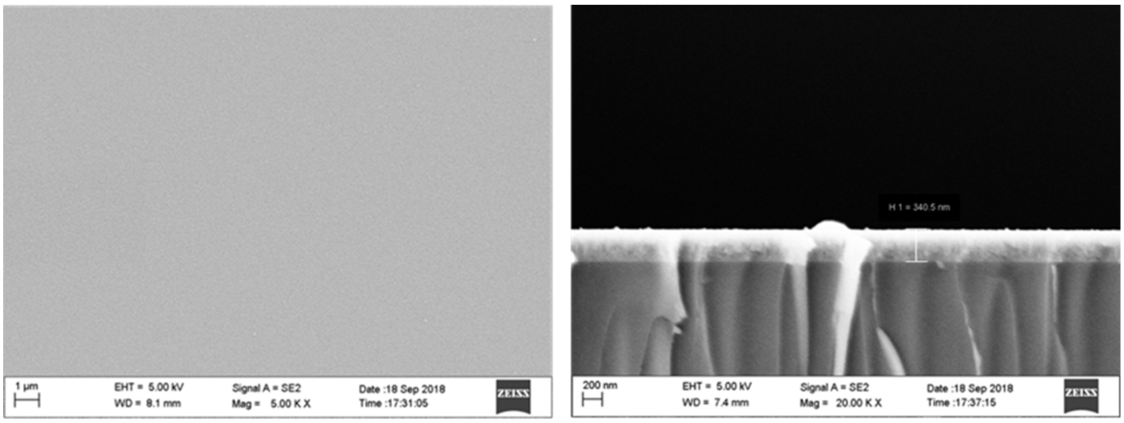

(a) (b)

Figure. S3. Image of sputtered SiO_2 of the Love wave device under SEM, a) Surface morphology; b) Cross-sectional views.

3. Demonstration of the immobilization of CEA antibody using a fluorescence microscope

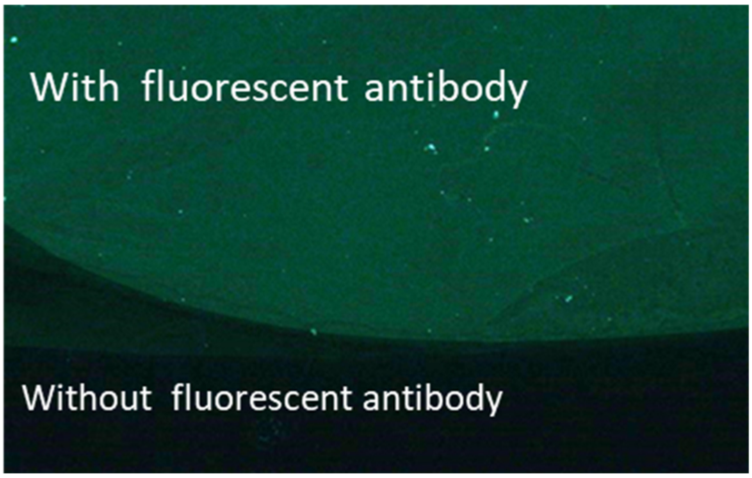

Figure. S4. Comparison of bound fluorescent capture CEA antibody and unbound region.
